# A novel *TAB2* nonsense mutation (p.S149X) causing autosomal dominant congenital heart defects: a case report of a Chinese family

**DOI:** 10.1186/s12872-019-01322-1

**Published:** 2020-01-20

**Authors:** Jia Chen, Huizhen Yuan, Kang Xie, Xinrong Wang, Linglong Tan, Yongyi Zou, Yan Yang, Lu Pan, Junfang Xiao, Ge Chen, Yanqiu Liu

**Affiliations:** 1 Prenatal Diagnosis Center, Jiangxi Provincial Maternal and Child Health Hospital, Nanchang, 330006 Jiangxi China; 2 Central Laboratory, Jiangxi Provincial Maternal and Child Health Hospital, Nanchang, 330006 Jiangxi China

**Keywords:** Congenital heart defects, Valvular anomalies, TAB2, Whole-exome sequencing

## Abstract

**Background:**

TAB2 is an activator of MAP 3 K7/TAK1, which is required for the IL-1 induced signal pathway. Microdeletions encompassing *TAB2* have been detected in various patients with congenital heart defects (CHD), indicating that haploinsufficiency of *TAB2* causes CHD. To date, seven variants within *TAB2* were reported associated with CHD, only two of them are nonsense mutations.

**Case presentation:**

Here we describe a three-generation Chinese family that included five CHD patients with heart valvular defects, such as mitral or tricuspid valves prolapse or regurgitation, and aortic valve stenosis or regurgitation. Our proband was a pregnant woman presenting with mitral, tricuspid, and aortic defects; her first child experienced sudden cardiac death at the age of 2 years. Whole-exome sequencing of the proband revealed a novel nonsense variant in *TAB2* (c.C446G, p.S149X), which results in the elimination of the majority of C-terminal amino acids of TAB2, including the critical TAK1-binding domain. The variant was identified in five affected patients but not in the eight unaffected family members using Sanger sequencing and was classified as “pathogenic” according to the latest recommendation on sequence variants laid out by the American College of Medical Genetics and Genomics and the Association for Molecular Pathology.

**Conclusion:**

We described a family with CHD caused by a novel *TAB2* nonsense mutation. Our study broadens the mutation spectrum of *TAB2*; to the best of our knowledge, this is the first report of a pathogenic mutation within *TAB2* in a Chinese population.

## Background

*TAB2* is a gene located on chromosome 6q25.1 [OMIM *605101] and encodes the TGF-β-activated kinase 1/MAP 3 K7 binding protein 2 (TAB2). As an adapter protein linking TGF-β-activated kinase 1 (TAK1/MAP 3 K7) and TNF receptor-associated factor 6 (TRAF6), TAB2 plays an essential role in the activation of JNK/NF-κB signaling induced by IL-1 [1]. Haploinsufficiency of *TAB2* has been linked to congenital heart defects (CHD) via mapping of the smallest overlapping region of various 6q25.1 microdeletions in different patients with CHD [2]. Further investigations have shown that TAB2 is expressed in embryonic cardiac tissues of both humans and zebrafish. Knocking down TAB2 in zebrafish embryos resulted in delayed epiboly progression, convergent extension defects during gastrulation (at approximately 12 h postfertilization), and severe heart failure 36–48 h postfertilization [2]. These findings indicate that TAB2 dysfunction causes CHD. Additionally, TAB2 mutations have also been found in patients with frontometaphyseal dysplasia (FMD), with apparently different phenotypes from CHD [3, 4].

**Fig. 1:**
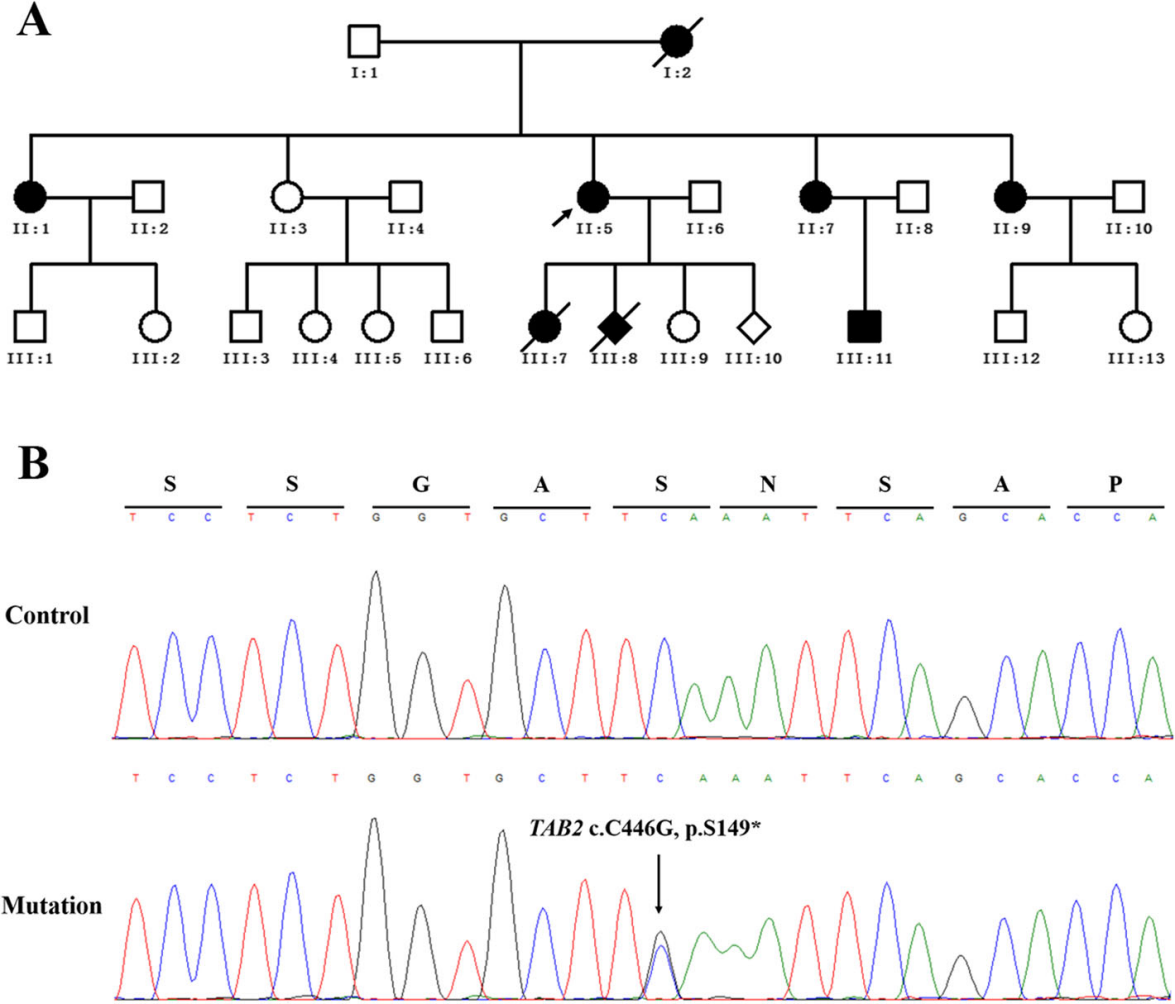
Pedigree of the CHD family and sequence results of the *TAB2* mutation. **a** All sampled subjects in the pedigree are identified by Roman numerals below the symbol. Arabic numbers denote each individual in a generation. Open symbols, unaffected; filled symbols, affected; symbols with a diagonal line, deceased subjects; squares, male; circles, female; diamond, fetus with unknown sex; arrow, the proband. **b** Sequence chromatogram indicates a C-to-G transition of nucleotide 466

**Fig. 2:**
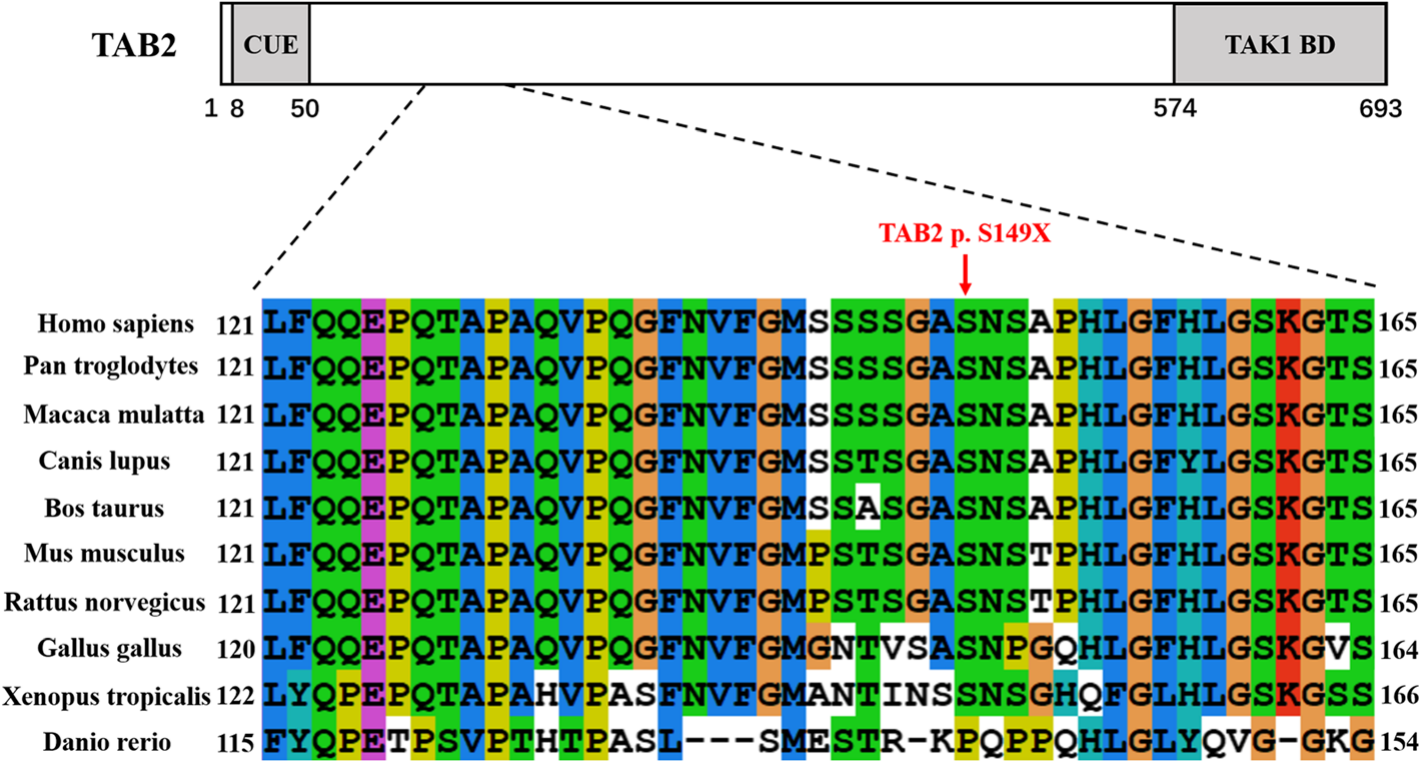
Analysis of protein domains and the mutation of TAB2. Schematic representation of TAB2 protein domains indicate an N-terminal coupling of ubiquitin conjugation to endoplasmic reticulum (CUE) domain and a C-terminal TAK1 binding domain (TAK1 BD). The affected amino acid S149, indicated by red arrow, is highly conserved in different species

According to the latest Human Gene Mutation Database (HGMD; http://www.hgmd.cf.ac.uk/ac/index.php), 21 deletions encompassing *TAB2* and seven disease-causing variants within the gene have been associated with CHD; only two of them are nonsense mutations. Here we examined a three-generation Chinese family including eight patients with CHD (Fig. 1a; Table 1). Heart valvular defects were detected in most affected patients by standard echocardiography. A novel heterozygous variant (c.C446G) in exon5 of *TAB2* was detected in the proband by Whole-Exome Sequencing (WES) and identified in all the surviving affected family members using Sanger sequencing (Fig. 1b; Table 1). This nonsense variant creates a premature stop codon at the 149th residue of TAB2 (p.S149X) and removes the majority of amino acids from the protein, including the TAK1-binding domain (TAK1 BD) (Fig. 2) [1]. This variant is classified as “pathogenic” as per the latest recommendation on sequence variants interpretation laid out by the American College of Medical Genetics and Genomics (ACMG) and the Association for Molecular Pathology (AMP) [5]. Our study expands the variant diversity of *TAB2* and provide more clinical symptoms of CHD patients caused by a TAB2 mutation in a Chinese population.

**Table 1:** Summary of the CHD family

CHD Family members	CHD	Age at diagnosis	Heart defects according to transthoracic echocardiogram	TAB2	
				DNA	Protein
II:1	Yes	39y	atrial septal aneurysm, left coronary artery dilation, mild aortic regurgitation	c.C446G	p.S149X
II:3	No	36y	–	–	–
II:5	Yes	31y	mild mitral and tricuspid regurgitation, mild aortic valve stenosis with aortic regurgitation	c.C446G	p.S149X
II:6	No	32y	–	–	–
II:7	Yes	30y	atrial septal aneurysm, mild mitral valves prolapse with mitral regurgitation, mild pulmonic regurgitation	c.C446G	p.S149X
II:8	No	31y	–	–	–
II:9	Yes	29y	atrial septal aneurysm, left atrial and ventricular dilatation	c.C446G	p.S149X
III:3	No	12y	–	–	–
III:4	No	11y	–	–	–
III:5	No	3y	–	–	–
III:9	No	2y	–	–	–
III:10	High risk	19w pregnancy	–	c.C446G	p.S149X
III:11	Yes	3y	mild left ventricular and right atrial dilation, mild mitral valves prolapse with mitral regurgitation	c.C446G	p.S149X
III:13	No	1y	–	–	–

*CHD* Congenital heart defect, *y* Years old, *w* Weeks; “-”, no defect or mutation detected

## Case presentation

### Clinical information

The study was approved by the institutional review board of Jiangxi maternal and child health hospital, Nanchang, China. All the enrolled subjects provided written informed consent. A family from the Jiangxi province, China, comprising 25 family members (11 males, 12 females, and 2 fetuses) across three generations (Fig. 1a) were included in the study. Our proband (II:5) was a 31-year-old female who was 15 weeks pregnant at her first visit to our hospital. She was slightly tachypneic and reported occasional fatigue since the age of 25 years old. She performed a transthoracic color echocardiogram examination at 30 years old and revealed mild aortic valve stenosis accompanied with mild mitral, tricuspid, and aortic regurgitation. She denied any surgical or pharmaceutical interventions. Her husband was 32 years old (II:6) and declared no cardiac symptoms or family history of heart diseases. Their first child (III:7) was a CHD girl with left and right ventricles dilatation. The girl then experienced sudden cardiac death (SCD) at 2 years of age. The second child (III:8) was detected with no heartbeat at 9 weeks of pregnancy using a Doppler fetal monitor, and subsequently, the pregnancy was terminated. The third child (III:9) was a 2-year-old girl with no symptoms of cardiac disorders. She was not diagnosed with any heart abnormalities using echocardiography.

The proband’s other family members were interviewed, and cardiac symptoms, such as tachypnea, shortness of breath, and fatigue, were reported. Four females and one male were identified with clinical symptoms (Fig. 1a). The proband’s mother (I:2) died in her late fifties due to sudden cardiac arrhythmia. Evident atrial septal aneurysms were detected in the proband’s sisters (II:1, II:7, and II:9) using echocardiography. Left coronary artery dilation was screened in the patient II:1 by echocardiography and then identified using coronary arteriography. Other cardiac anomalies, such as mild mitral valves prolapse with mitral and pulmonic regurgitation, and left atrial and ventricular dilatation, were found in II:7 and II:9, respectively. III:11 was a 3-year-old boy who was diagnosed with mild left ventricular and right atrial dilation as well as mild mitral valves prolapse with mitral regurgitation using echocardiography. Transabdominal fetal echocardiography was performed on the proband’s fetus (III:10) at 15 weeks of pregnancy, and no cardiac abnormality was detected. The heart defects in the individuals enrolled in this study are summarized in Table 1.

### Mutation detection

An autosomal dominant inheritance pattern was suggested on the basis of vertical transmission of CHD in the family. Peripheral blood was obtained from 13 family members (II:1, II:3, II:5, II:6, II:7, II:8, II:9, III:3, III:4, III:5, III:9, III:11, and III:13). Genomic DNA was extracted from the peripheral blood lymphocytes using the QIAamp DNA blood mini kit (Qiagen).

To determine the causative mutation in the family, WES of the proband (II:5) was performed as previously described with minor modifications [6]. Two micrograms of the genomic DNA from the proband II:5 was used for human whole-exome analysis with paired-end-sequencing at 100× resolution. Libraries were constructed using the SureSelect Human All ExonV7 kit (Agilent Technologies, USA) and sequenced on the Illumina HiSeq 2500 platform (Illumina, San Diego, CA), as per the manufacturer’s instructions. The reads were aligned to the human reference genome (University of California Santa Cruz, UCSC hg19) using SOAPaligner. Single-nucleotide polymorphism (SNP) and indel (insertion or deletion) identification was performed using SAMtools and/or the Genome Analysis Toolkit (GATK), and SNPs with a read depth > 4 and quality > 20 were used for subsequent analyses. SNPs and indels were annotated using SeattleSeq annotation. Known polymorphisms in the dbSNP (https://www.ncbi.nlm.nih.gov/snp/) (minor allele frequency, > 0.01) and 1000 genomes (https://www.ncbi.nlm.nih.gov/variation/tools/1000genomes/) (genotype frequency, > 0.005) databases as well as synonymous single-nucleotide variants and variants not located in exonic or splicing regions were excluded. Pathogenicity of the obtained variants was predicted using Polyphen-2 (http://genetics.bwh.harvard.edu/pph2/index.shtml) [7], SIFT (https://sift.bii.a-star.edu.sg/) [8], and MutationTaster (http://www.mutationtaster.org/) [9].

**Fig. 3:**
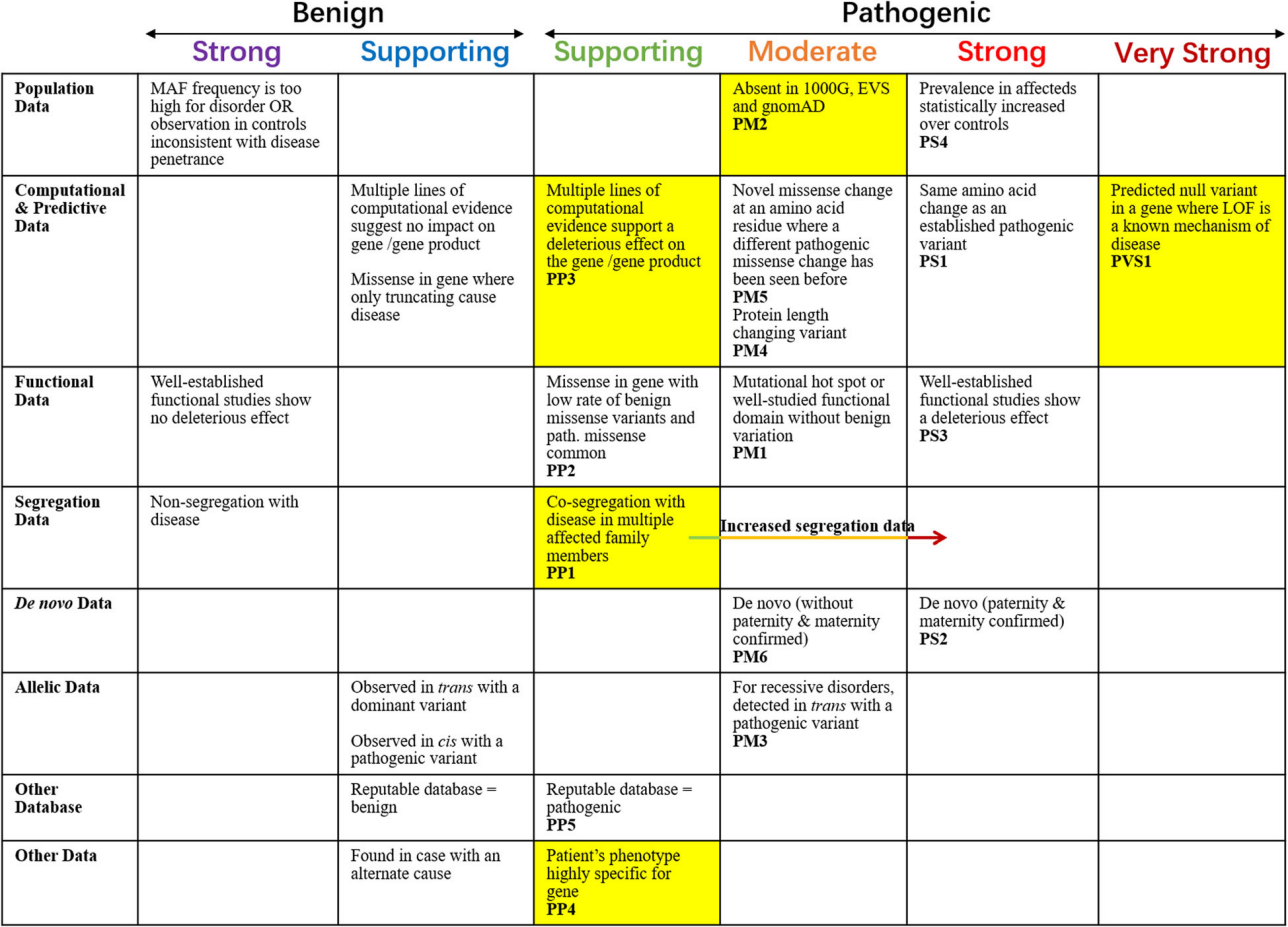
Variant assessment of S149X in TAB2 according to the recommendations of the ACMG and the AMP. Criteria fulfilled by this variant are indicated in yellow. This figure has been adapted from Richards et al. [5]

Approximately 99.71% of the sequencing reads were mapped to the human genome hg19, with mean 186.76× sequencing depth. A heterozygous variant in *TAB2* (c.C446G, NM_015093.5), which results in a premature stop codon with early termination of protein translation at residue 149 (p.S149X), passed the filtering criteria. Using Sanger sequencing, this variant was detected in all the affected family members (II:1, II:5, II:7, II:9, and III:11); it was absent in the unaffected individuals (II:3, II:6, II:8, III:3, III:4, III:5, III:9, and III:13) (Fig. 1b). The variant, with no record in 1000 genomes, the Exome Variant Server (EVS; http://evs.gs.washington.edu/EVS/), the Genome Aggregation Database (gnomAD; http://gnomad.broadinstitute.org/), and the HGMD databases, was not detected in our 531 control cohorts using high-resolution melting analysis (SsoFast EvaGreen, Bio-Rad). TAB2 protein sequences (from *Danio rerio* to *Homo sapiens*) were obtained from the protein database of the National Center for Biotechnology Information (NCBI, https://www.ncbi.nlm.nih.gov/). Alignments of TAB2 protein family members using cluster 2.0 software [10] revealed that the affected amino acid is evolutionarily conserved (Fig. 2). According to the latest recommendation by ACMG and AMP on sequence variants interpretation, the c.C446G variant in *TAB2* would be classified as “pathogenic”, having met the requirements of fulfilling one very strong (PVS1), one moderate (PM2) and three supporting (PP1, PP3 and PP4) criteria (Fig. 3) [5].

A prenatal molecular genetic diagnosis for the fetus (III:10) was recommended for evaluating the risk for CHD. With complete informed consent of the proband (II:5) and her husband (II:6), a prenatal diagnosis of the fetus (III:10) was performed at 19 weeks of pregnancy. The amniocytes was obtained by amniocentesis, and fetal genomic DNA (III:10) was extracted using the QIAamp DNA mini kit (Qiagen). A heterozygous *TAB2* c. C446G variant was detected, indicating that the fetus had a high risk for CHD.

## Discussion and conclusions

TAB2 is a 693-amino acid protein and plays an important role in the IL-1 signaling pathway and cardiac development. In the present study, a nonsense mutation in *TAB2* was identified in five living patients with cardiac defects in a Chinese family. Most patients in the family presented mild clinical symptoms, such as slightly tachypnea and occasional fatigue, except for the proband’s first girl child (III:7) who had left and right ventricular dilatation and suffered a SCD at 2 years old. The dilation of left ventricle is a hallmark of dilated cardiomyopathy (DCM) and may contribute to the unexpected death of patient III:7. DCM is defined by the presence of left ventricular dilatation and contractile dysfunction and serves as a leading cause of SCD, especially in childhood [11, 12]. Patients carrying *TAB2* mutations have been associated with DCM. A 7-month-old girl with *TAB2* c.1168delT mutation was diagnosed with DCM and conducted a heart transplant surgery at 9 months old, then died at 2.5 years old [13]. DCM was also detected in a 60-year-old brother and a 48-year-old sister who were both carrying a *TAB2* c.1398dupT mutation [14]. In addition, arrhythmia, another risk factor of SCD, had been reported in patients with *TAB2* mutations [2, 14].

The heart defects detected using transthoracic echocardiography in the affected members were heterogeneous and mainly involved the heart valves. The proband was diagnosed with mitral, tricuspid, and aortic valve regurgitation. Aortic, pulmonic, and mitral valve dysfunctions were observed in two of her sisters (II:1 and II:7) and one of her nephews (III:11), respectively. In addition, three of the proband’s sisters (II:1, II:7, and II:9) had atrial septal aneurysm. Dilation in the coronary artery or heart chambers was detected in patients II:1, II:9, and III:11.

Mutations disrupting *TAB2* are associated with heart valvular defects. Several studies have reported that CHD patients with 6q24-q25 microdeletions, containing the *TAB2* locus, experience valve anomalies, such as valvular stenosis, mitral or aortic regurgitation, and atrial or ventricular septal defects [2, 14–17]. Similar valvular defects have also been detected in patients with missense, nonsense, and small insertion or deletion mutations within *TAB2*. A c.622 C > T (p.P208S) and a c.688 C > A (p.Q230K) mutation in *TAB2* were identified in a woman with aortic regurgitation and a man with bicuspid aortic valve, respectively [2]. WES of a male child with polyvalvular syndrome revealed a c.1491 T > A nonsense mutation (p.Y497X) in *TAB2* [18]. Pulmonary artery aneurysm, moderate mitral regurgitation, and mild tricuspid regurgitation were discovered in a family with a c.1039 C > T nonsense (p.R347X) *TAB2* mutation [19]. A c.1398dupT mutation in *TAB2* was confirmed in a family with polyvalvular heart disease [14]. Recently, a girl with a de novo *TAB2* c.1168delT mutation was diagnosed with dilated cardiomyopathy at 7 months of age; she underwent a heart transplant after 2 months and died at 2.5 years of age [13].

Apart from cardiac defects, TAB2 mutations are associated with connective tissue disorders. The male child with CHD due to the TAB2 p.Y497X mutation also had hypotonia, myopia, soft pale skin, joint hypermobility, and mild facial dysmorphism [18]. Similar clinical features were observed in the family with the *TAB2* c.1398dupT mutation [14]. However, no abnormality in the connective tissues was observed in the patients in our study. The family carrying the TAB2 p.R347X nonsense mutation did not show any connective tissue disorders either [19]. These findings suggest clinical heterogeneity of extracardiac tissues in patients with TAB2 mutations. In addition, a c.1705 G > A mutation (p.E569K) and a c.1619 A > G mutation (p.Q540R) in *TAB2* were detected in patients with FMD, a progressive sclerosing skeletal dysplasia that affects the long bones and the skull. Different from the loss-of-function mutations involved in CHD, these TAB2 mutations cause FMD through a gain-of-function mechanism [3, 4].

A detailed understanding of the loss-of-function mutations of TAB2 that cause heart valvular defects remains unclear. The endothelial-to-mesenchymal transition (EndMT) process which endothelial cells migrate from the endocardial layer into the cardiac jelly and acquire mesenchymal characteristics to form the cardiac valves is essential in cardiac valvular development. This process is mainly regulated by the TGF-β signaling [20]. Disturbances in the TGF-β pathway undermine EndMT [21, 22] and cause valvular diseases, such as mitral valve degeneration and myxomatous atrioventricular valve diseases [23, 24]. TAB2 plays an important role in the TGF-β pathway by phosphorylating the TAK1/MAP 3 K7 protein, which then activates NF-κB and MAPK signaling [25]. TAB2 mutations may cause valvular defects by disrupting the EndMT process controlled by the TGF-β pathway.

In summary, this study describes a three-generation Chinese family with a history of CHD. In this family, five living patients mainly presented with heart valvular defects. Whole-exome and direct sequencing were used to trace the genetic cause of the disease. We identified the first *TAB2* mutation (c.C446G, p.S149X) in a Chinese population. Molecular prenatal diagnosis was performed for the proband’s fetus after the mutation was suggested to be pathogenic as per the latest recommendation on sequence variants interpretation laid out by the ACMG. The mutation may disturb cardiac valvular development by impeding the EndMT process regulated by the TGF-β pathway. Our study broadens the mutation spectrum of the *TAB2* gene and implies that TAB2 plays a crucial role in the EndMT process.

## Data Availability

We did not use new software, databases, or applications/tools in the manuscript, all results and figures have already provided in the manuscript.

## Abbreviations

ACMG: The American College of Medical Genetics and Genomics
AMP: The Association for Molecular Pathology
CHD: Congenital heart diseases
DCM: Dilated cardiomyopathy
EndMT: Endothelial-to-mesenchymal transition
EVS: Exome variant server database
FMD: Frontometaphyseal dysplasia
GATK: Genome analysis toolkit
gnomAD: Genome aggregation database
HGMD: Human gene mutation database
indel: Insertion or deletion
NCBI: National Center for biotechnology information
SCD: Sudden cardiac death
SNP: Single-nucleotide polymorphism
TAK1 BD: TAK1-binding domain
WES: Whole-exome sequencing
